# Robust Prediction of Immune Checkpoint Inhibition Therapy for Non-Small Cell Lung Cancer

**DOI:** 10.3389/fimmu.2021.646874

**Published:** 2021-04-13

**Authors:** Jiehan Jiang, Zheng Jin, Yiqun Zhang, Ling Peng, Yue Zhang, Zhiruo Zhu, Yaohui Wang, De Tong, Yining Yang, Jianfei Wang, Yadong Yang, Kui Xiao

**Affiliations:** ^1^ Department of Pulmonary and Critical Care Medicine, University of South China Affiliated Changsha Central Hospital, Changsha, China; ^2^ Research Institute, GloriousMed Clinical Laboratory (Shanghai) Co., Ltd, Shanghai, China; ^3^ Department of Respiratory Disease, Zhejiang Provincial People's Hospital, Hangzhou, China; ^4^ Tongji Medical College, Huazhong University of Science & Technology, Wuhan, China; ^5^ Department of Pulmonary and Critical Care Medicine, The Second Xiangya Hospital, Central South University, Changsha, China

**Keywords:** immunotherapy, non-small cell lung cancer, biomarkers, immune pathway, neural network, prognosis

## Abstract

**Background:**

The development of immune checkpoint inhibitors (ICIs) is a revolutionary milestone in the field of immune-oncology. However, the low response rate is the major problem of ICI treatment. The recent studies showed that response rate to single-agent programmed cell death protein 1 (PD-1)/programmed cell death-ligand 1 (PD-L1) inhibition in unselected non-small cell lung cancer (NSCLC) patients is 25% so that researchers defined several biomarkers to predict the response of immunotherapy in ICIs treatment. Common biomarkers like tumor mutational burden (TMB) and PD-L1 expression have several limitations, such as low accuracy and inadequately validated cutoff value.

**Methods:**

Two published and an unpublished ICIs treatment NSCLC cohorts with 129 patients were collected and divided into a training cohort (n = 53), a validation cohort (n = 22), and two independent test cohorts (n = 34 and n = 20). We identified six immune-related pathways whose mutational status was significantly associated with overall survival after ICIs treatment. Then these pathways mutational status combined with TMB, PD-L1 expression and intratumor heterogeneity were incorporated to build a Bayesian-regularization neural networks (BRNN) model to predict the ICIs treatment response.

**Results:**

We firstly proved that TMB, PD-L1, and mutant-allele tumor heterogeneity (MATH) were independent biomarkers. The survival analysis of six immune-related pathways revealed the mutational status could distinguish overall survival after ICIs treatment. When predicting immunotherapy efficacy, the overall accuracy of area under curve (AUC) in validation cohort reaches 0.85, outperforming previous predictors in either sensitivity or specificity. And the AUC in two independent test cohorts reach 0.74 and 0.80.

**Conclusion:**

We developed a pathway-model that could predict the efficacy of ICIs in NSCLC patients. Our study made a significant contribution to solving the low prediction accuracy of immunotherapy of single biomarker. With the accumulation of larger data sets, further studies are warranted to refine the predictive performance of the approach.

## Introduction

Immunotherapy is emerging as a beneficial tool for cancer treatment by activating the immune system to produce antitumor effects (1). Recently, the most advanced approach to therapeutically utilize the antitumor activity is via immune checkpoint inhibitors (ICIs) (2). Immune checkpoint inhibitors work by releasing a natural brake on patient's immune system so that immune cells called T cells to recognize and attack tumors (3). Among the ICIs, programmed cell death protein 1(PD-1)/programmed cell death-ligand 1(PD-L1) and cytotoxic T-lymphocyte-associated protein 4(CTLA-4) inhibitors showed promising therapeutic outcomes, and some have been approved for numerous cancer therapy, such as melanoma, renal cell carcinoma (RCC), and non-small cell lung cancer (NSCLC) (4, 5). However, ICIs are not universally effective for all patients, and many patients fail to respond to ICIs due to intrinsic resistance or have an initial response followed by disease progression due to acquired resistance (6). For example, response rates to single-agent PD-1/PD-L1 inhibition in unselected patients with melanoma, NSCLC, and RCC are 40% (7, 8), 25% (8, 9), and 19% (10), respectively (11). To identify patients who are more likely to respond to PD-1/PD-L1 blockade as well as other immunotherapeutics, researchers defined several biomarkers to predict the response of immunotherapy in cancer treatment. The commonly used biomarkers include tumor mutational burden (TMB) and PD-L1 expression (11, 12). Patients with a higher TMB or higher PD-L1 expression have a higher likelihood of immunotherapy response. Another novel statistical value, mutant-allele tumor heterogeneity (MATH), has been documented that is not only as a measure of intratumor genetic heterogeneity but also can be used as a biomarker to predict the response of treatment for patients (13–16). In addition, recent studies have shown that some pathways, such as IFN-gamma, NF-κb, and Wnt, are cancer-related immune-regulation pathway, which may be potential indicators to explore the effect of immunotherapy (17–20).

Nevertheless, it has been documented that the available biomarkers have several limitations (21, 22), such as low accuracy, and inadequately validated cutoff value, and previous studies only use one or two of them independently in immunotherapy prediction (23). Therefore, we developed a pathway-model that included TMB, PD-L1, MATH, and immune-related pathway to predict the efficiency of ICIs, especially in NSCLC, which is the leading cause of cancer-related morality worldwide (24). The pathway-model did not only have a high accuracy in published cohorts but also be proven to have an effective prediction ability in GloriousMed cohort with 20 NSCLC patients. This study made a significant contribution to solving the low prediction accuracy of immunotherapy of single biomarker.

## Materials and Methods

### GloriousMed Cohort

Twenty patients with non-small cell lung cancer treated with PD-1/PD-L1 inhibitors in The Second Xiangya Hospital, Central South University who had genomic profiling of whole exome sequencing (WES) before treatment were included in our GloriousMed cohort (**Supplementary Table S1**).

TMB was defined as the total number of somatic mutations per exome in megabases. PD-L1 staining was evaluated centrally by IHC using 22C3 antibody and an automated staining procedure developed by Dako. The percentage of PD-L1 expression was scored by a qualified pathologist in samples with a minimum of 100 viable tumor cells.

Objective response was assessed by investigator-assessed RECIST 1.1 criteria every 6 weeks (two cycles of ICB administration). The complete response (CR) or partial response (PR) was considered as responders, whereas patients with stable disease (SD) or progressive disease (PD) were considered as non-responders.

All patients collection and usage were in accordance with the principles of the Declaration of Helsinki and approved by the Institution Review Board of The Second Xiangya Hospital, Central South University. The written informed consent for sample acquisition was obtained from all patients. All data were deidentified.

### Public Cohorts

Three independent public cohorts including Hellmann cohort (25), Rizvi cohort (26), and Samstein cohort (27) were also used in this study. The data for the three independent cohorts were retrieved from published articles (**Supplementary Table S2**). Hellmann cohort included 75 NSCLC patients treated with combined PD-1 and CTLA-4 blockade. Rizvi cohort included 34 NSCLC patients that treated with pembrolizumab. The Samstein cohort contained 1,662 patients received immunotherapy from 11 different cancers.

### WES Sequencing

DNA was extracted from FFPE-fixed tumor tissue using QIAamp DNA FFPE Tissue Kit (Qiagen), and Genomic DNA (gDNA) was extracted from white blood cells using the Blood Genomic DNA Mini Kit (Cwbiotech). Integrated DNA Technologies's xGen Exome Research Panel v1.0 according to the standard procedures (IDT) were used to capture whole exome. For each sample, 200 to 500 ng FFPE DNA or 500 ng gDNA was then used for library preparation and quantification guided by KAPA Hyper Prep protocols (KAPA). Libraries were then purified by AMPure XP (Beckman) and quantified by Qubit™ dsDNA HS Assay Kit (Thermo Fisher). Final library was sequenced on the Illumina Novoseq6000 (PE150). Sequencing adapters were trimmed by Trimmomatic from the raw data (28). The reads after adapter trimming were then aligned with the human reference genome hg19 by BWA (29). Duplicated reads were removed by Picard. Mapped reads were also realigned to the genome by Genome Analysis Tool Kit. Somatic mutations were called by Mutect2 with a paired workflow. Variants were then annotated by ANNOVAR and self-development code (30). An in-house script was used to verify the human identity concordance of paired samples. Somatic mutations were filtered with the following rules: (1) base quality value ≥20; (2) mutation reads depth ≥10; (3) variant allele frequency ≥5%; (4) reads supporting variation <4 and frequency <2% in normal, tumor abundance/normal abundance ≥8; (5) no strand bias (GATK parameter FS > 60 for SNP and FS >200 for indel); (6) discard synonymous mutations.

### Quantitative and Statistical Analyses

TMB and PD-L1 expression of Hellmann cohort and Rizvi cohort were retrieved from published articles. MATH was calculated through R package maftools for GloriousMed, Hellmann and Rizvi cohorts (31). Correlation among TMB, MATH, and PD-L1 expression (%) were examined by the Pearson rank correlation method. Correlation between TMB or MATH and grouped PD-L1 expression were examined by the Wilcoxon signed-rank test.

The overall survival (OS) was defined from the start of ICIs treatment until death due to any cause. And the progression-free survival (PFS) was defined as the time from the start of ICIs treatment until disease progression. Of notes, the Samstein cohort merely published OS data and Rizvi cohort provided PFS data. The Kaplan-Meier method was used to estimate OS or PFS, and the log-rank test was used to compare the survival curves. All tests with a p value ≤ 0.05 were considered statistically significant.

### Immune-Related Pathway Selection

The detailed profiles of genes involved in HRR, MMR, BER, JAK, MAPK, PI3K, NF-κB, and Wnt pathways were listed in **Supplementary Table S3**. At first, mutational status of aforementioned six immune-related pathways in every sample was classified into two categories: the first one assigned with 0 (no non-synonymous mutation) and the second with 1 (at least one non-synonymous mutation). Then, DDR pathway mutation status of each sample was classified into three groups based on the mutational status of HRR, MMR, and BER. “N” represented no mutation in HRR, MMR, or BER, “C” was stood for co-mutation between HRR and MMR or BER, and “S” was other cases. In addition, the mutational status of PI3K, JAK, and NF-κB were integrated as one variable by summing the mutational status.

### Model Construction

Three models were constructed, one model with TMB, PD-L1 expression, MATH, and immune-related pathways, called “pathway-model”; a second with TMB, PD-L1 expression, and MATH, called “tri-model”; the last one, called “bivariate-model”, with TMB and PD-L1 expression (**Table 1**). Both TMB and MATH were z-score normalized. PD-L1 expression was stratified as 0% (Z), 1%-49% (L), ≥50% (H), or unknown (N). And immune-related pathways were processed according to Immune-Related Pathway Selection. All of the models were trained via Bayesian Regularized Neural Networks (BRNN) algorithm using corresponding variables with 2 layers and default hyperparameters from R package caret (32), and the resampling method “boot” was used to choose the optimal model. The cutoff value of single-factor variable, TMB, PD-L1 expression and MATH was estimated by BRNN algorithm as well. Fifty-three patients of the Hellmann cohort were used as the training set, and remaining 22 patients were validation set. Rizvi cohort and GloriousMed cohort were processed as above description and were used as testing cohort.

**Table 1:** Models and variables.

Model	Variable
Bivariate-model	TMB and PD-L1 expression
Tri-model	TMB, PD-L1 expression and MATH
Pathway-model	TMB, PD-L1 expression, MATH and immune-related pathways

TMB, tumor mutational burden; PD-L1, programmed cell death-ligand 1; MATH, mutant-allele tumor heterogeneity.

### Model Performance Evaluation

Receiver operating characteristic (ROC) curves were constructed with the predictor estimated from each of the previous models and single-factor variables with roc function of R package pROC (33). Benefit probability of each patient was extracted from prediction results, and DCB/NDB information was provided by the cohorts. Differences between DCB and NDB with benefit probability were examined by the Wilcoxon signed-rank test.

### Comprehensive Analysis of TCGA LUAD and LUSC Cohorts

The clinical information, RNA expression, mutational status and protein array of The Cancer Genome Atlas Lung Adenocarcinoma (TCGA LUAD) and Lung Squamous Cell Carcinoma (LUSC) patients were retrieved from TCGA database. The patients with EGFR exon 18–21 mutations and ALK gene fusions were filtered to avoid make a disturbance for the analysis. In the signature score analysis, the expression of genes in a signature was normalized in the form of fragments per kilobase of exon model per million mapped fragments (FPKM). Then, a principal component analysis (PCA) was performed, and PC1 was extracted to serve as gene signature score (34). The 18 signatures and their gene sets were summarized from published papers (34–38). The significantly differential expression analysis was based on DESeq2 (39). The row counts of LUAD and LUSC patients were used as input for DESeq2. The differential expression genes were defined as the genes with absolutely log2Foldchange ≥ 1 and p-value ≤ 0.05. The oncoplot of top 30 mutated genes were drawn by using R package maftools (31).

## Results

### TMB, PD-L1 Expression, and MATH Are Independent Variables

**Figure 1:**
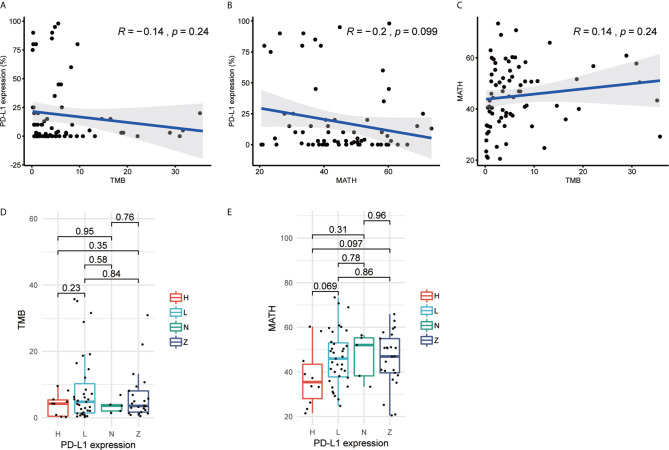
Tumor mutational burden (TMB), programmed cell death-ligand 1 (PD-L1) expression and mutant-allele tumor heterogeneity (MATH) are independent from each other in Hellmann cohort. **(A)** Scatterplot between TMB and PD-L1 expression (%). **(B)** Scatterplot between MATH and PD-L1 expression (%). **(C)** Scatterplot between TMB and MATH. **(D)** Boxplot of TMB and PD-L1 expression. **(E)** Boxplot of MATH and PD-L1 expression. The R value of **(A–C)** represents Pearson correlation coefficient.

The previous studies documented that higher TMB or PD-L1 expression correlated with better outcomes as compared with lower TMB or PD-L1 expression (11, 12, 25, 40). However, in 70 of 75 patients from Hellmann cohort who had all three biomarkers data, correlation between TMB and PD-L1 expression was not significant (R=-0.14, p-value=0.24). TMB of some patients was more than 10 but PD-L1 expression was less than 25% (**Figure 1A**). The results might reveal the biomarkers were not consistent in response prediction of ICIs treatment. In the meantime, the novel biomarker MATH was not significantly correlated with PD-L1 expression (R = −0.2, p-value = 0.099) or TMB (R = 0.14, p-value = 0.24) as well (**Figures 1B, C** **)**. We further explored the correlation between stratified PD-L1 expression and TMB or MATH by stratifying PD-L1 expression as 0% (Z), 1% to 49% (L), ≥50% (H), and unknown (N). Neither MATH nor TMB showed a significant difference with any PD-L1 expression groups (**Figures 1D, E** **)**. The Rizvi and GloriousMed cohort showed the consistent correlation results as well (**Supplementary Figure 1**). This lack of correlation suggested that TMB, PD-L1 expression, and MATH are independent predictive measures of response to ICIs treatment, and a robust model should be constructed to unify these variables.

### Mutational Status of Immune-Related Pathway Can Act as Candidate Biomarkers

**Figure 2:**
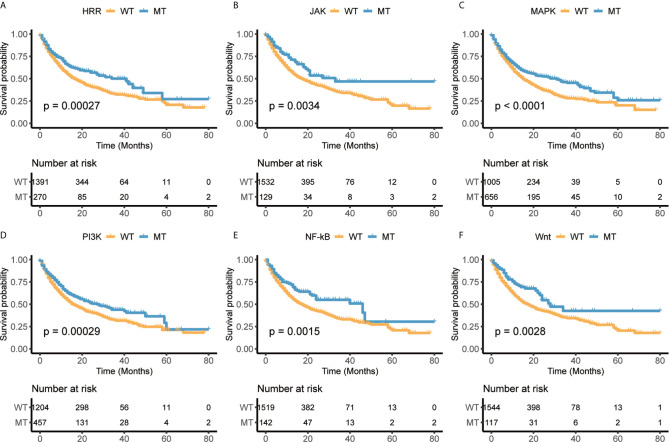
The mutational status of selected immune-related pathways are significantly associated with overall survival (OS) in Samstein cohort. **(A)** Homologous recombination repair (HRR). **(B)** Janus kinase (JAK). **(C)** Mitogen-activated protein kinase (MAPK). **(D)** Phosphoinositide 3-kinase (PI3K). **(E)** Nuclear factor kappa-light-chain-enhancer of activated B cells (NF-κB). **(F)** Wnt.

A prior study has shown that co-mutation information of DNA damage response (DDR) pathway can be used as a predictor of response to immune checkpoint blockade, and the mutation of the DDR solved the problem of difficulty in determining an optimal TMB threshold (22). This finding provided a new way to predict the response of immunotherapy. Besides DDR pathway, we selected six pathways, homologous recombination repair (HRR), Janus kinase (JAK), mitogen-activated protein kinase (MAPK), phosphoinositide 3-kinase (PI3K), and nuclear factor kappa-light-chain-enhancer of activated B cells (NF-κB), Wnt, through literature survey, which are associated with tumor immunity or immunotherapy escape (41, 42). We also collected the mutational status of these pathways from Samstein cohort treated with ICIs (27) and explored its correlation with the overall survival (OS). The results showed that patients with mutations in any of six pathways had better survival than those without mutation (**Figure 2**). Furthermore, the results also revealed the selected pathways could be used as biomarkers to distinguish the prognosis for ICIs treatment.

### Pathway Model Is the Best Model to Predict the Efficiency of ICIs Treatment

**Figure 3:**
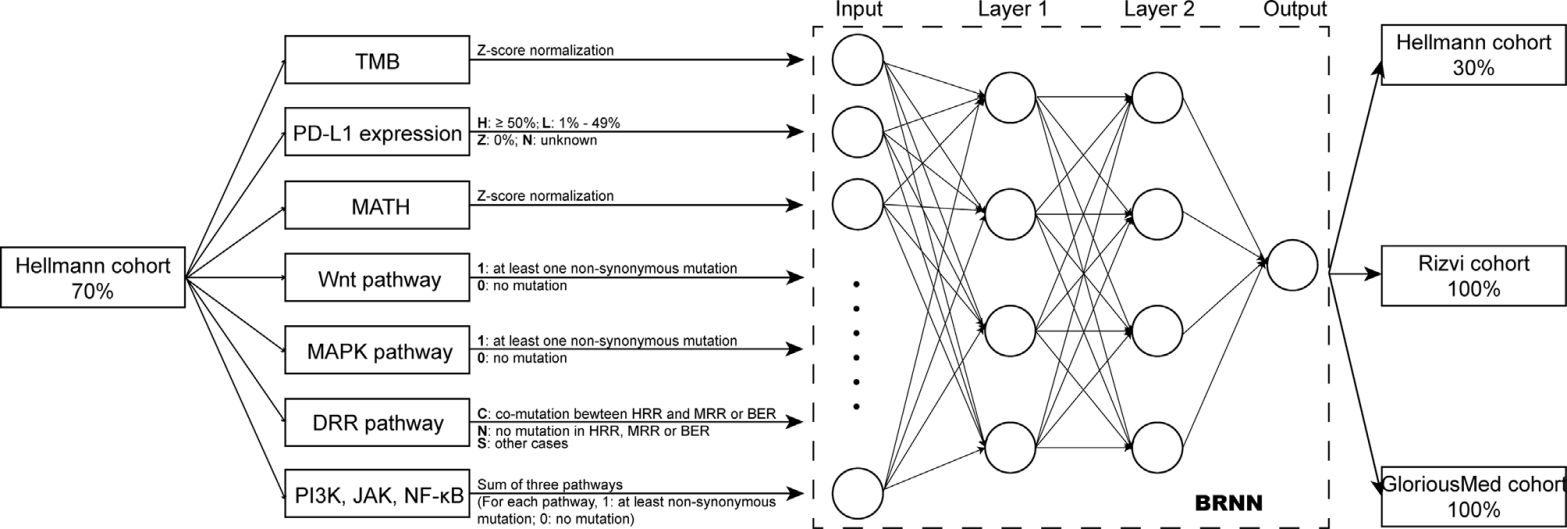
Overview of the model design. Pathway-model was constructed and trained by 70% Hellmann cohort. Then, the predictor was tested in one validation cohort (the remaining 30% of Hellman cohort) and two independently testing cohorts (100% of Rizvi cohort and 100% of GloriousMed cohort).

**Figure 4:**
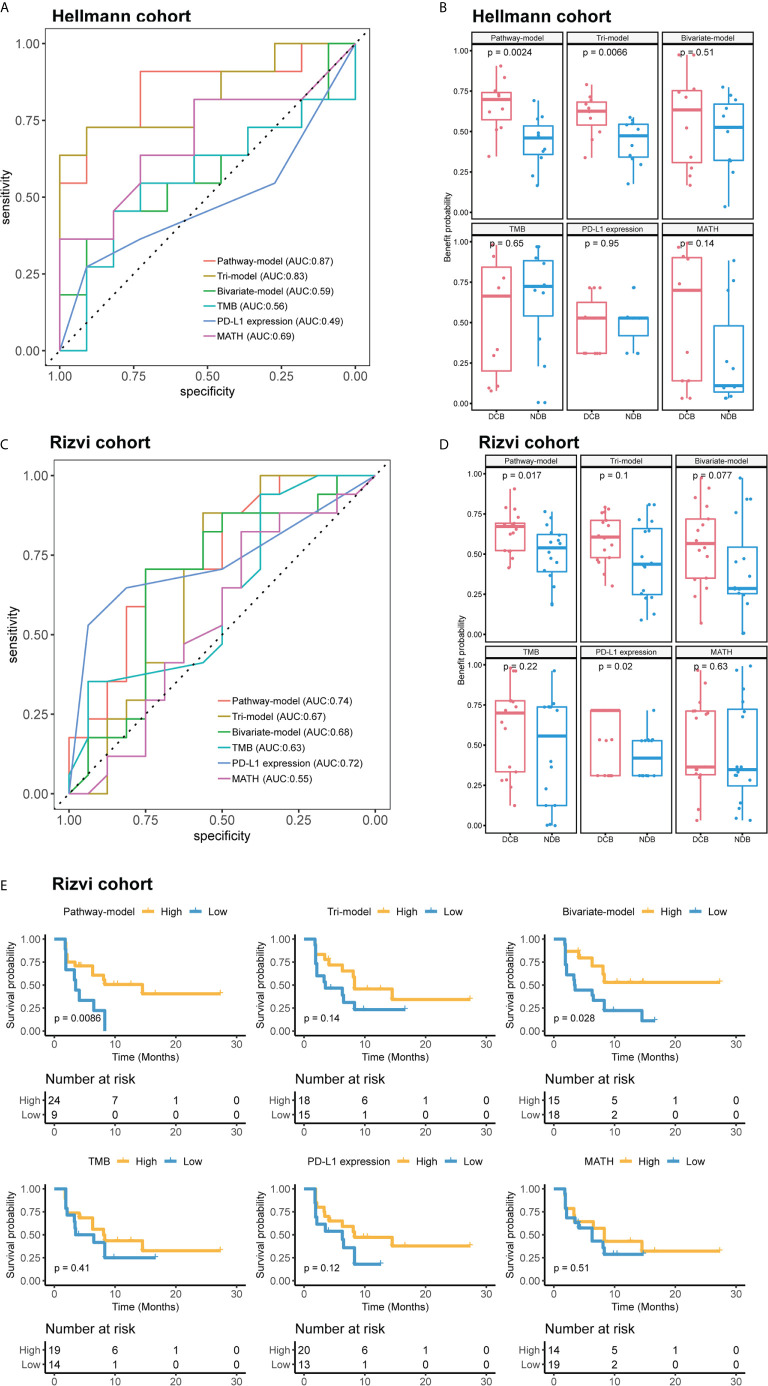
The performance comparison different models and single-factor variables of in validation cohort (Hellmann cohort) and independent test cohort (Rizvi cohort). **(A)** Receiver operating characteristic (ROC) curves of different models. **(B)** Benefit probability and risk of patients in different response groups. **(C)** ROC curves of different models. **(D)** Benefit probability and risk of patients in different response groups. **(E)** Survival analysis based on different models and single-factor variables, time was progression-free survival (PFS). Patients of **(A, B)** were from Hellmann cohort, and patients of **(C–E)** were from Rizvi cohort.

We extracted 70% patients from Hellmann cohort, which totally included 75 NSCLC patients, as training data set (25) and the rest 30% patients were used to validate the models. Three different models were trained by using the training data set with different variables and were adjusted with clinical benefit as outcomes (**Table 1**). The pathway-model contains seven variables, including TMB, PD-L1 expression, MATH and the mutational status of six immune-related pathways (**Figure 3**). The mutational status of JAK, MAPK, and PI3K was integrated into one variable to improve the prediction accuracy. ROC curves based on the predictor for each of the three models estimated on Hellmann cohort (22 patients) were available and the results showed that the pathway-model was more predictive than other two models (AUC is 0.87, 0.83, and 0.59 for pathway-, tri-, and bivariate-model). The AUC of pathway-model was higher than single-factor variables containing TMB, PD-L1 expression, and MATH as well (AUC is 0.56, 0.49, and 0.69 for TMB, PD-L1 expression, and MATH) (**Figure 4A** and **Table 2**). We also checked the prediction benefit probability, a quantitative output generated from the model which represents the likelihood of immunotherapy response, of each patient compared with real clinical benefit information among three models. The benefit probability generated from pathway-model and tri-model are significantly higher in DCB group than in NDB group (p-value is 0.0024 for pathway-model and 0.0066 for tri-model), however, the median benefit probability of pathway-model (0.70) was higher than tri-model (0.46). The difference of benefit probability was not significant in other models and single factors (**Figure 4B**).

**Table 2:** Performance of models in three cohorts.

	Pathway-Model	Tri-model	Bivariate-model	TMB	PD-L1	MATH
Hellmann cohort	0.87	0.83	0.59	0.56	0.49	0.69
Rizvi cohort	0.74	0.67	0.68	0.63	0.72	0.55
GloriousMed cohort	0.80	0.47	0.64	0.65	0.78	0.46

TMB, tumor mutational burden; PD-L1, programmed cell death-ligand 1; MATH, mutant-allele tumor heterogeneity.

We further tested the predictive ability of pathway-model in Rizvi cohort (26), consisting of 34 NSCLC patients treated with pembrolizumab, with all predictive variables and clinical benefit information available. The results showed that pathway-model could more accurately predict the clinical benefit of ICIs than other two models and single-factor variables (AUC is 0.74 for pathway-model, 0.67 for tri-model, 0.68 for bivariate-model, 0.63 for TMB, 0.72 for PD-L1 expression, and 0.55 for MATH) (**Figure 4C** and **Table 2**). The benefit probability of patients in DCB and NDB groups was significantly different as well (p-value is 0.0017, **Figure 4D**). The survival analysis indicated that the high benefit probability group also showed a better PFS (**Figure 4E**).

### Pathway Model Can Precisely Predict the Response of ICIs Treatment in GloriousMed Cohort

**Figure 5:**
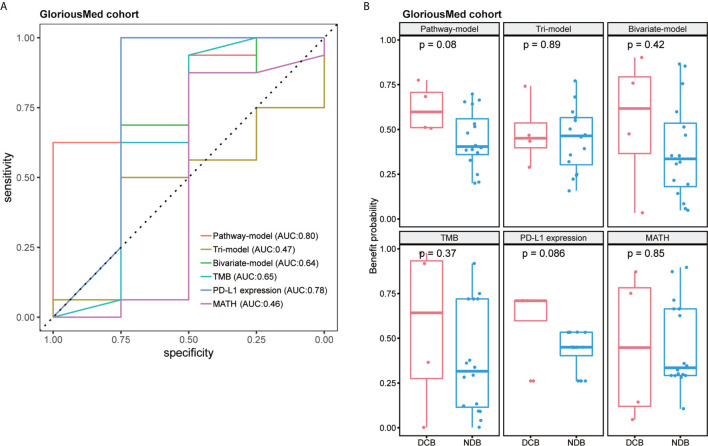
The performance comparison of different models in GloriousMed cohort. **(A)** Receiver operating characteristic (ROC) curves of different models. **(B)** Benefit probability and risk of patients in different response groups.

Finally, we tested pathway-model in GloriousMed cohort with 20 NSCLC patients, who were treated by PD-1/PD-L1 inhibitors (**Supplementary Table S1**). The accuracy of pathway-model was much higher than tri-model and bivariate-model (AUC is 0.80 for pathway-model, 0.47 for tri-model and 0.64 for bivariate-model) (**Figure 5A** and **Table 2**). Even though, the benefit probability was not significantly different between DCB and NDB group (p-value is 0.08 for pathway-model), all DCB patients have a predictive benefit probability higher than 0.5 (**Figure 5B**). Thus, pathway-model can be generalized in clinical to improve the prediction accuracy of the response to immunotherapy.

### Comprehensive Analysis With TCGA NSCLC Cohort Imply that High Benefit Probability Patients Is Associated With Immune Response

**Figure 6:**
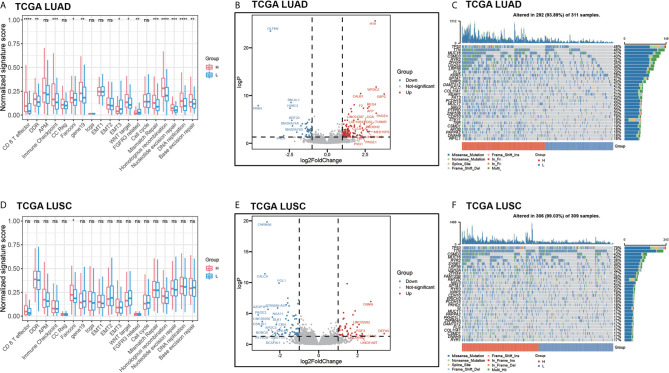
The comprehensive analysis between high benefit probability group and low benefit probability group in The Cancer Genome Atlas Lung Adenocarcinoma (TCGA-LUAD) and Lung Squamous Cell Carcinoma (LUSC) cohort. High and low group were stratified based on median of benefit probability of the patients through pathway-model. **(A)** The significant signature scores of 18 gene sets in LUAD cohort. **(B)** Differential expression genes in LUAD cohort. **(C)** Oncoplot of top 30 mutated genes in LUAD cohort. **(D)** The significant signature scores of 18 gene sets in LUSC cohort. **(E)** Differential expression genes in LUSC cohort. **(F)** Oncoplot of top 30 mutated genes in LUSC cohort.

We predicted the benefit probability of TCGA LUAD and TCGA LUSC cohorts without EGFR exon 18-21 mutations and ALK gene fusions patients in immunotherapy with pathway-model and classified patients to two groups at the median cut-point. Then, we calculated signature scores of 18 gene sets with principle component analysis (PCA) method. In TCGA LUAD cohort, thirteen signatures are significantly different between high benefit probability group and low probability group (**Figure 6A**). In consideration of TMB, and mutational status of DDR and Wnt pathways are included in prediction model, the benefit probability difference in DDR, WNT target, DNA repair–related signatures and cell cycle were expected. The signature score of CD 8 T effector and Immune Checkpoint were higher in high probability group than in that of low group, while the signature score of EMT3 and FGFR3 related was lower in high probability group (**Figure 6A**). However, in LUSC cohort, we did not find significant difference between high and low benefit probability groups as LUAD cohort (**Figure 6D**).

Furthermore, we analyzed the differential expression genes between high benefit probability groups and low group in LUAD and LUSC respectively (**Figures 6B, E**, **Supplementary Table S4**). There are 153 differential expression genes (106 up-regulated) in LUAD, including *AFP* and *G6PC*, which related to P53 downstream pathway and FOXO pathway. In LUSC, there are 120 differential expression genes (50 up-regulated) including *FGF3* and *DLK1*, which related to FGFR pathway and NOTCH pathway. Apart from that, part of the top 30 mutated genes, such as *KRAS* and *PTPRD*, have different mutation pattern between high benefit probability group and low group, as well as between LUAD and LUSC (**Figures 6C, F** **)**.

Above all, the comprehensive analysis of TCGA LUAD and LUSC cohorts imply that high benefit probability patients from pathway-model is associated with immune response.

## Discussion

Immune checkpoints inhibitors (ICIs), such as PD-1 and PD-L1, have revolutionized the treatment of many cancers, including NSCLC. However, how to select patients most likely to benefit from immunotherapy is the current leading challenge in the field. Previous ICIs-related studies preferred to use several single biomarkers, respectively, to predict the prognosis of immunotherapy (25, 26). Our study constructed a robust pathway-model based on deep learning approach, which included two common biomarkers, TMB, PD-L1 expression, a recent developed intratumor heterogeneity evaluation value MATH and potential marker-immune-related pathways. To the best of our knowledge, this is the first study to combine mutational status of pathways and common biomarkers for efficacy of prediction in NSCLC. Not only the ROC curves but also the significant difference of benefit probability from our predictor between DCB and NDB showed that our model had high accuracy in both training and test NSCLC data sets. The comparison among our pathway-model, tri-model, bivariate-model, and single-factor variables showed that our pathway-model had the highest accuracy in predicting the response to ICIs treatment. We found that tri-model with MATH had a lower AUC than bivariate-model without MATH in Rizvi and GloriousMed cohort. However, there is no denying that MATH did not improve the efficacy in distinguishing DCB and NDB patients in Rizvi and GloriousMed cohort in tri-model compared with bivariate-model. And pathway-model with MATH is the most stable model compared to other models and single factor variables. A recent study has shown that the integration of TMB and MATH forms a predictive marker for the response of ICIs treatment in melanoma (16), and another study has also revealed that intratumoral heterogeneity (MATH is an indicator of intratumoral heterogeneity) can be used as a biomarker to predict the response of ICIs treatment in NSCLC (15). Moreover, we found that the common biomarkers were not significant correlation according to the Pearson correlation coefficient, and the accuracy of each single-factor variable was lower than the pathway-model or tri-model. It might indicate there was a great synergy among these biomarkers. When we grouped the patients at the median of benefit probability generated from pathway-model, the PFS time was significantly different between high and low group, specifically patients with high benefit probability were more likely to have longer PFS time. These results suggested that besides the ability of response prediction of ICIs treatment, benefit probability is also associated with the prognosis of NSCLC patients. In addition to, the prediction results of GloriousMed cohort prove that our pathway-model can effectively predict the benefit probability of ICIs treatment and can be generalized in clinical to provide some reference during the treatment.

Furthermore, the enrichment analysis of 18 immune-related gene sets in TCGA LUAD and LUSC cohort suggested that our model might reveal the possible mechanism of the immune phenotype of tumors. Previous studies have proven that CD8 cell play a central role in immunity to cancer through their capacity to kill malignant cells, EMT-related genes may contribute to tumor immune escape, and FGFR mutated cases have a more deserted immune phenotype than the wild type (43–46). Our immune infiltration analysis also showed that the high benefit probability group of LUAD cohort had higher CD8 T effector scores. However, the significant difference of signature scores between high benefit probability group and low group were only found in TCGA LUAD cohort, but not in TCGA LUSC cohort. It is implied that the underlying immune response mechanism may be different between LUAD and LUSC. The differential expression genes in LUAD and LUSC are not complete same. P53 downstream pathway and FOXO pathway may be enriched in LUAD due to the up-regulation genes *AFP* and *G6PC*. P53 signaling pathway has been known as an important pathway in immune response, for example, it can function in immune cells including myeloid and T cells (47). Previous study has shown that FOXO pathway can be a target in tumor drug development (48). In LUSC, two differential expression genes, *FGF3* and *DLK1* are related two different pathways, FGFR pathway and Notch pathway. The enrichment of FGFR pathway implies a desert-immune subtype and high tumor purity of LUSC (45). Notch pathway can control the fate of various T cell type and myeloid cells that down-regulated DLK1 might influence the immune cells (49). The different regulated pathways between LUAD and LUSC may be one of the reasons of different immune response mechanism. In LUAD cohort, the mutation ratio of *KRAS*, an oncogene which leads to immune escape in the tumor microenvironment (50), and *PTPRD*, which affects the tumor proliferation (51), were higher than LUSC also suggests the difference immune response mechanisms. All above inference is based on naïve treatment public cohort, the exact mechanism would still to be explored with treatment samples. Except that, the probability of some differential expression genes, such as *MUC2*, *CLCA1*, *REG4*, and *FGF3* can be used as prognostic biomarkers in NSCLC is worth exploring because they have been reported as a biomarkers in other cancers as well (52–55).

There were limitations in our study that should be acknowledged. First, patients in the training cohort were treated with Nivolumab Plus Ipilimumab, and the model generated from which may be distracted in predicting patient in test cohort treated with Pembrolizumab or Tislelizumab due to pharmaceutical and medication differences. Second, the PD-L1 expression was quantified with different antibodies in training and validation cohort. Also, in the exploring cohort in TCGA data set, the PD-L1 expression was quantified using reverse phase protein array. The platform discordant of PD-L1 quantification may impair the power of our prediction model. Besides, due to the limitation of the training data sets, it is difficult to get a satisfactory model. Also, there are other features that are not incorporated into our model due to unavailability in either training or validation cohort, such as immune phenotype, which is known to affect the immunotherapy efficacy. In future studies, we will include more patients and features to guarantee the training process and the clinical practice of the predicting ICIs treatment efficacy in NSCLC patients.

## Data Availability Statement

The raw data supporting the conclusions of this article will be made available by the authors, without undue reservation.

## Ethics Statement

The studies involving human participants were reviewed and approved by The Second Xiangya Hospital, Central South University. The patients/participants provided their written informed consent to participate in this study.

## Author Contributions

KX, YiY, JJ, and JW conceived and designed the project. YiZ, DT, and LP prepared and collected the data. ZJ, YuZ, YiZ, and ZZ contributed to analysis and interpretation. KX, ZJ, JJ, and YaY drafted the manuscript. KX, YW, JJ, JW, and YaY performed the quality assessment and revised the manuscript. All authors contributed to the article and approved the submitted version.

## Funding

This study was supported by Scientific Research Project of Human Provincial Health Commission (No. 202103020704).

## Conflict of Interest

Authors ZJ, YiZ, YiY, JW and YaY were employed by GloriousMed Clinical Laboratory (Shanghai) Co., Ltd. 

The remaining authors declare that the research was conducted in the absence of any commercial or financial relationships that could be construed as a potential conflict of interest.
