# Supplementary material for: Robust Prediction of Immune Checkpoint Inhibition Therapy for Non-Small Cell Lung Cancer
Source: Front Immunol. 2021 Apr 13;12:646874. doi: 10.3389/fimmu.2021.646874 (PMC8076602; doi:10.3389/fimmu.2021.646874)
Supplement: Supplementary file 1 [file Image_1.pdf]

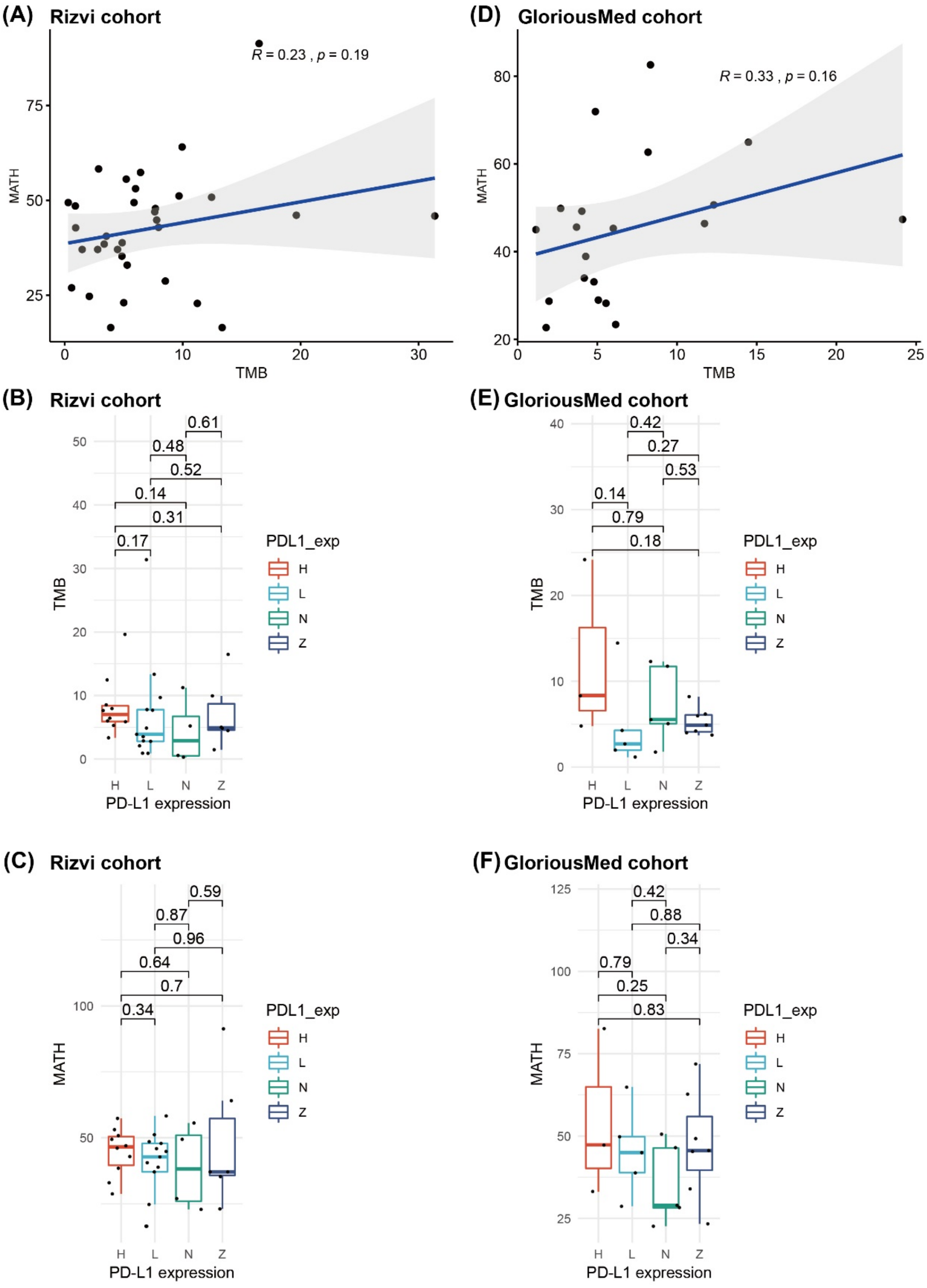

Supplementary Figure 1. TMB, PD-L1 expression and MATH are independent from each other in Rizvi and GloriousMed cohorts. **(A)** Scatterplot between TMB and MATH in Rizvi cohort. **(B)** Boxplot of TMB and PD-L1 expression in Rizvi cohort. **(C)** Boxplot of MATH and PD-L1 expression in Rizvi cohort. **(D)** Scatterplot between TMB and MATH in GloriousMed cohort. **(E)**

Boxplot of TMB and PD-L1 expression in GloriousMed cohort. **(F)** Boxplot of MATH and PD-L1 expression in GloriousMed cohort. The R value of **(A)** and **(D)** represents Pearson correlation coefficient.
