# Supplementary material for: Robust Prediction of Immune Checkpoint Inhibition Therapy for Non-Small Cell Lung Cancer
Source: Front Immunol. 2021 Apr 13;12:646874. doi: 10.3389/fimmu.2021.646874 (PMC8076602; doi:10.3389/fimmu.2021.646874)
Supplement: Supplementary file 2 [file Table_1.docx]

**Supplementary Table S1. Patient characteristics in the GloriousMed cohort.**

| **Characteristics** | **No. (%)** |
| --- | --- |
| **No. of patients** | 20 (100) |
| **PFS, months (median)** | 4.96 |
| **Best overall response** |  |
| PR | 4 (20) |
| SD | 11 (55) |
| PD | 5 (25) |
| **Clincial Benefit** |  |
| DCB | 4 (20) |
| NDB | 16 (80) |
| **PD-L1 expression** |  |
| ≥50% | 3 (15) |
| ≥1% | 5 (25 |
| <1% | 7 (35) |
| Unknown | 5 (25) |
| **TMB (median)** | 4.97 |
| **MATH (median)** | 45.43 |
| **Mutation** |  |
| EGFR (18-21 exon) | 0 (0) |
| ALK | 0 (0) |

Abbreviations: PFS, progression-free survival; PR, partial response; SD, stable disease; PD, progression disease; DCB, durable clinical benefit; NDB, no durable benefit; PD-L1, programmed death-ligand 1; TMB, tumor mutation burden; MATH, mutant-allele tumor heterogeneity; EGFR, epidermal growth factor receptor; ALK, anaplastic lymphoma kinase.
