# Supplementary material for: Robust Prediction of Immune Checkpoint Inhibition Therapy for Non-Small Cell Lung Cancer
Source: Front Immunol. 2021 Apr 13;12:646874. doi: 10.3389/fimmu.2021.646874 (PMC8076602; doi:10.3389/fimmu.2021.646874)
Supplement: Supplementary file 3 [file Table_2.docx]

**Supplementary Table S2. Patient characteristics in the public cohorts.**

| **Characteristics** | **No. (%)** |  |
| --- | --- | --- |
|  | **Hellmann** | **Rizvi** |
| **No. of patients** | 75 (100) | 34 (100) |
| **PFS, months (median)** | 5.42 | 6.3 |
| **Best overall response** |  |  |
| CR | 4 (5) | 0 (0) |
| PR | 20 (27) | 14 (41) |
| SD | 27 (36) | 17 (50) |
| PD | 24 (32) | 3 (9) |
| **Clincial Benefit** |  |  |
| DCB | 24 (32) | 14 (41) |
| NDB | 51 (68) | 20 (59) |
| **PD-L1 expression** |  |  |
| ≥50% | 10 (13) | 10 (29) |
| ≥1% | 35 (47) | 14 (41) |
| <1% | 25 (34) | 6 (18) |
| Unknown | 5 (6) | 4 (12) |
| **TMB (median)** | 4.16 | 5.29 |
| **MATH (median)** | 44.89 | 42.88 |
| **Mutation** |  |  |
| EGFR (18-21 exon) | 0 (0) | 0 (0) |
| ALK | 9 (12) | 1 (3) |

Abbreviations: PFS, progression-free survival; OS, overall survival; CR, complete response; PR, partial response; SD, stable disease; PD, progression disease; DCB, durable clinical benefit; NDB, no durable benefit; PD-L1, programmed death-ligand 1; TMB, tumor mutation burden; MATH, mutant-allele tumor heterogeneity; EGFR, epidermal growth factor receptor; ALK, anaplastic lymphoma kinase.
