# Supplementary material for: Robust Prediction of Immune Checkpoint Inhibition Therapy for Non-Small Cell Lung Cancer
Source: Front Immunol. 2021 Apr 13;12:646874. doi: 10.3389/fimmu.2021.646874 (PMC8076602; doi:10.3389/fimmu.2021.646874)
Supplement: Supplementary file 4 [file Table_3.docx]

**Supplementary Table S3. Genes involved in immune-related pathways.**

| **Pathway** | **Gene** |
| --- | --- |
| **HRR** | BLM, BRCA1, BRCA2, DMC1, EME1, EME2, GEN1, HFM1, MRE11, MUS81, NBN, PPP4C, PPP4R1, PPP4R2, PPP4R4, RAD50, RAD51, RAD51B, RAD51C, RAD51D, RAD52, RAD54B, RAD54L, RAD54L2, RDM1, RECQL, RECQL4, RECQL5, RMI1, RMI2, RPA1, RPA2, RPA3, SEM1, SLX1A, SLX4, PPP4R3A, PPP4R3B, SPO11, TOP3A, TOP3B, WRN, XRCC2, XRCC3 |
| **MMR** | EXO1, HMGB1, LIG1, MLH1, MLH3, MSH2, MSH3, MSH4, MSH5, MSH6, PCNA, PMS1, PMS2, POLD1, POLD2, POLD3, POLD4, RFC1, RFC2, RFC3, RFC4, RFC5, RPA1, RPA2, RPA3 |
| **BER** | APEX1, APLF, APTX, CCNO, FEN1, HMGB1, LIG1, LIG3, MBD4, MPG, MUTYH, NEIL1, NEIL2, NEIL3, NTHL1, OGG1, PARP1, PARP2, PARP3, PARP4, PCNA, PNKP, POLB, POLD1, POLD2, POLD3, POLD4, POLE, POLE2, POLE3, POLE4, POLL, SMUG1, TDG, TDP1, UNG, XRCC1 |
| **PI3K** | PIK3A-G, AKT1, EIF4EBP1, EIF4E, RPS6KB1, MTOR, FRAP, PDK1, MDM2 |
| **Wnt** | APC, APC1, APC2, AXIN1, AXIN2, GSK3B, BACH1, NFAT5, ATF3, SNAI1, SNAI2, SNAI3, PPARA, PPARD, PPARG, RXRA, RXRB, RXRG, TCF3, SOX11, PKN1, SOX7, E2F1 |
| **JAK** | SOCS1, PTPN6, PTPN11 |
| **MAPK** | NPM, ALK, RAC1, RAC2, CDC42, SOS1, KRAS, HRAS, NRAS, RAF1, BRAF, ARAF, SHC1, SHC2, SHC3, SHC4, GRB2, ERK1, ERK2, JNK1, JAK2, JAK3, MAPK1, MAPK2, MAPK3, MERTK, ATF2, ETS1, ELK1, MEF2 |
| **NF-kB** | NFKB1, NFKB2, TRADD, TRAF2, TRAF3, TRAF6, MSK1, MSK2, MAP3K7, TAB1, TAB2, TAB3, ATF1, ATF2, CHUK, IKBKB, IKBKG, ATM, BFL1, REL, RELA, RELB, BCL2 |
