# Supplementary material for: Robust Prediction of Immune Checkpoint Inhibition Therapy for Non-Small Cell Lung Cancer
Source: Front Immunol. 2021 Apr 13;12:646874. doi: 10.3389/fimmu.2021.646874 (PMC8076602; doi:10.3389/fimmu.2021.646874)
Supplement: Supplementary file 5 [file Table_4.docx]

**Supplementary Table S4. Differential expression genes in TCGA LUAD and LUSC cohorts.**

| **Cohort** | **Regulation** | **Genes** |
| --- | --- | --- |
| **LUAD** | Up-regulated | CLCA1, PAGE1, EPHA8, PAX2, BPIFB2, AFP, TF, GABRP, KCNK10, PAGE4, VGLL1, CALB1, NEFM, SLC1A6, SULT2A1, ATP4A, HOXA1, NEURL1, LGI1, VTN, ANXA10, APOA5, KNG1, NPPB, SPINK4, NKX2-4, FAM83C, HOXD11, UNC13A, H19, G6PC, BEX1, REG4, EPHA7, CGA, PAX3, NR5A1, SERPINA10, CYP1A1, ASTN1, CPB1, TMPRSS11D, SST, IGF2BP1, LRRC38, EN1, ALPP, TAFA4, MAGEC3, SOHLH1, CRABP1, BPIFB6, KCNK9, CT55, BNC1, KRT13, DSCAM, WFDC5, APOF, PCSK1, SOX11, TH, F2, ADGRD2, TPRXL, GREM2, PENK, OR6T1, CCNYL2, HS6ST3, MPPED1, NPSR1, SLC30A10, CYP2B6, LRRTM3, RNU5A-1, TCEAL5, LOC100130449, MED15P9, MRPL23-AS1, MIR5689HG, PSG1, ERVH48-1, LOC105373352, LINC01287, PRSS56, SCARNA10, HOXA11-AS, PAQR9-AS1, TUNAR, LOC654780, PRODH2, LINC00958, HOXA10-AS, LINC01606, LINC01419, LOC101926892, TAS2R30, SALL3, GPR142, XKR7, LOC112267895, LINC02582, IGFL2-AS1, NEFL, H2BC10 |
|  | Down-regulated | TDRD1, OLFM4, KRT23, FOLR3, APOBEC1, MMP8, SOHLH2, KCNK17, CRP, CHI3L1, EMX1, RHCG, CHODL, SLC1A7, FRMPD4, KRT20, MUCL1, SAA1, FMR1NB, SHISA3, UCN3, PSAPL1, CCDC190, ATP13A5, BNIP5, ABCA4, MUC2, RNU4-1, SNORA74A, SPDYC, CD177, PRSS1, LINC00443, PRB4, LOC100996404, PAGE2B, GSTA2, BMPR1B-DT, SNORA74D, SAA2-SAA4, H2BC14, H4C6, H2AC14, H2BC3, H2AC4, H4C1, RN7SL3 |
| **LUSC** | Up-regulated | CLCA4, CAPN6, BPIFB2, PROKR2, MSLN, DKK4, LIM2, LCT, GDPD2, TNNT3, CA6, RAX, DSG1, CRNN, CPLX2, SYT8, DEFA5, KRT4, PSG6, CSN3, KRT9, MS4A10, FAM9B, DYNAP, ADIPOQ, SRARP, SPRR4, SIX6, FGF3, SAMD7, KRT77, NXF4, NPIPB13, EPS8L3, MUC2, UCA1, TDRD15, LINC01529, PLSCR5, LINC00442, LINC01166, LINC00458, LINC00330, LINC01258, LOC100130268, LINC02404, LINC01467, LINC02582, LINC00906, BANCR |
|  | Down-regulated | TKTL1, C8B, NMRK2, LAMB4, NEFH, FGL1, NPTX2, NOBOX, AMBP, BST1, APOC3, CALCA, NEUROG3, PAX1, CSN1S1, CNMD, SIX3, ASCL1, BCL2A1, DPEP3, S100A7, CHRNA6, CRH, CPB1, MAGEA8, NAA11, PAGE5, TFF2, AIRE, AZGP1, IZUMO2, ZMAT4, GFI1B, KCNJ4, FOXI1, ADAM18, SOX14, GP2, HSD17B13, HOXB9, OBP2B, INSM1, TDRD12, HS3ST4, KCNB2, S100A7A, MRGPRE, KRT76, GALNT17, DLK1, VSTM2B, ASCL4, HEPACAM2, IL17REL, HMX3, C9orf129, ST8SIA6-AS1, FAM205A, VIT, AZGP1P1, SOX1-OT, DCAF8L1, LINC00200, PAGE2, ARHGDIG, SIRLNT, LOC441601, KHDC1L, TMEM238L, GRIN2B |
